# Prevalence and distribution of occult fractures on skeletal surveys in children with suspected non-accidental trauma imaged or reviewed in a tertiary Dutch hospital

**DOI:** 10.1007/s00383-020-04706-z

**Published:** 2020-06-26

**Authors:** Marie-Louise H. J. Loos, Tayiba Ahmed, Roel Bakx, Rick R. van Rijn

**Affiliations:** 1 Amsterdam UMC, Department of Paediatric Surgery, Emma Children’s Hospital, University of Amsterdam and Vrije Universiteit Amsterdam, Meibergdreef 9, 1105AZ Amsterdam, The Netherlands; 2 Amsterdam UMC, Department of Radiology and Nuclear Medicine, Emma Children’s Hospital, University of Amsterdam, Meibergdreef 9, 1105AZ Amsterdam, The Netherlands; 3 Department of Forensic Medicine, Section On Forensic Paediatrics, Netherlands Forensic Institute, PO Box 24044, 2490 AA The Hague, The Netherlands

**Keywords:** Non accidental injury, Paediatric injury, Fractures and dislocations, x-ray, Skeletal survey

## Abstract

**Purpose:**

The purpose of the study was to determine the rate of occult fractures (without clinical symptoms) per presenting clinical injury i.e., children presenting with a fracture, bruise, abusive head trauma and the types of fracture most likely to be found, in a series of infants and young children suspected of being victims of NAT.

**Methods:**

Skeletal surveys done between 2008 and 2018 of children (< 5 years) were retrospectively analyzed. Both radiographs of admitted children and reassessment images from all over the country were included and reviewed by a forensic paediatric radiologist. Deceased children were excluded. Variables as gender, age, initial clinical injury and occult fractures were collected. Occult fractures on the follow-up skeletal survey were collected.

**Results:**

A total of 370 skeletal surveys of 296 children were included. Median age was 22 weeks (IQR 11–48), there were 172 (58%) boys. A total of 195 occult fractures were detected in 111 (32%) children. Occult fractures were detected in 37/126 (29%) children with fracture as presenting symptom, 33/90 (37%) children with head trauma and 26/50 (52%) children with bruises. Rib (*n* = 56, 50%) and lower leg (*n* = 40, 36%) fractures were most detected.

**Conclusion:**

Occult fractures were detected in 32% of the children. Occult fractures were most prevalent if the initial clinical injury suggestive for NAT to request skeletal survey was a bruise, abusive head trauma or fracture.

## Introduction

Children who are suspected of non-accidental trauma (i.e., physical child abuse or injuries due to neglect) are usually referred and evaluated by a multidisciplinary team, the child abuse and neglect team (CAN—team). The CAN—team advices—in accordance with guidelines—which diagnostic tests should be used in order to identify injuries, such as a full head-to-toe examination to detect bruises and a skeletal survey to detect occult fractures (fractures without any apparent clinical symptoms).

According to the guideline ‘The radiological investigation of suspected physical abuse in children’ of the Royal College of Radiologists and Society and College of Radiographers, a skeletal survey should be performed in all children younger than 2 years old when non-accidental trauma is suspected [1, 2]. A skeletal survey may be indicated for older children under specific circumstances e.g., when the child has a developmental disorder or is immobilized. A follow-up skeletal survey is obtained approximately 2 weeks after the first skeletal survey, in order to detect occult fractures that were missed. Especially rib fractures can easily be missed when there is no callus present [3, 4]. An overview of the radiographs of the skeletal survey are provided in an e-supplement [1, 5].

The prevalence of occult fractures varies between different age groups and presenting clinical injury. The highest prevalence of occult fractures is found in young children < 12 months old 13–26% versus 6–18% in older children (≥ 24 months old) [6]. Although several studies addressed the prevalence of occult fractures, to date very few reported the distribution of these occult fractures. The overall aim of our research was to help health professionals involved in the treatment of trauma in children to diagnose NAT, by describing the clinical features of NAT most commonly associated with occult fractures and the types of fracture most likely to be detected on skeletal surveys.

The purpose of this study is to answer the following research questions: (1) What is the distribution of occult fractures on the skeletal survey? (2) What is the prevalence of occult fractures per clinical injury?

## Methods

The reports of skeletal surveys of all children < 5 years old performed between July 2008 and July 2018 were retrospectively reviewed. Skeletal surveys were either requested at Emma Children’s Hospital, University of Amsterdam, Amsterdam UMC, or were referred from other hospitals in the Netherlands for a second opinion by our paediatric radiologist (RRvR) specialized in forensic pediatric radiology. The Emma Children’s Hospital is a tertiary care university children’s hospital designated as a level I trauma center which employs paediatricans specialized in child abuse. RRvR has 17 years of working experience as a paediatric radiologist and 10 years as a forensic paediatric radiologist and is employed at both our hospital as well as the Netherlands Forensic Institute.

The Medical Ethics Review Committee of the Academic Medical Center Amsterdam reviewed this study (reference number W18_105 #18.134 d.d. 6th of april 2018) and, with a waiver of informed consent requirements, confirmed that the Medical Research Involving Human Subjects Act (WMO) does not apply and an official approval of this study was not required.

### Inclusion and exclusion criteria

All skeletal surveys of children younger than 5 years old were included in this study, this included the initial skeletal survey and, if available, the follow-up. Only skeletal surveys that were requested for the evaluation of child abuse were included. Initial clinical injuries suggestive for NAT for performing the skeletal survey were divided into different categories according to the information attached to the request: suspected fracture(s), suspected abusive head trauma, bruise(s), a non-accidentally injured sibling or twin and a final category of remaining or unknown. Skeletal surveys of deceased children were excluded.

Second opinions were requested because of various reasons, among others to confirm the existence of occult fractures. The results of the skeletal survey can have major implications, which is why images are often sent to a specialist to assess the images.

### Data extraction and outcomes

The primary outcome of this study is the distribution of occult fractures on skeletal survey of children. Secondary outcomes are the frequency of detected occult fractures, the frequency and distribution of occult fractures per initial clinical injury suggestive of NAT and whether skeletal surveys were performed confirm the official protocol as stated by the Royal College of Radiologists of the United Kingdom [2].

One researcher (ML) anonymized the reports of eligible skeletal surveys i.e., removed personal data of the patient. We extracted demographic information on the children, all occult fractures, the initial clinical injury suggestive of NAT for requesting the skeletal survey (clinical injury), whether a follow-up skeletal survey was performed and if there were occult fractures, and at last whether skeletal surveys were performed in accordance with the protocol. The index fracture was used to define the clinical injury and was not accounted as occult fracture, i.e., if the index fracture was the only fracture, we concluded that the skeletal survey was negative. We did not have access to medical files or initial reports of included patients, all relevant information was provided in the request of the skeletal survey, as well as in house requests as second opinions.

All data were collected via a web-based electronic case record form using Castor EDC [7], which is compliant with the Good Clinical Practice Guidelines.

### Statistical analyses

The frequency and distribution of occult fractures are analyzed and expressed as total count and percentage. Whether skeletal surveys were performed according to protocol was extracted as a binary outcome and is expressed as total count and percentage. Demographics are expressed as count and percentage or median and interquartile range. The data were analyzed with IBM SPSS Statistics V.24.0 or higher (IBM Corp., Armonk, NY, USA).

## Results

In total 370 skeletal surveys of 296 children were included. There were 296 primary skeletal surveys and 74 (25%) follow-up surveys. The children had a median age of 22 weeks (IQR 11–48). The demographics are presented in Table 1.

**Table 1:** Demographic information

Age in months^a^	Boy	Follow-up	Second opinion	Occult fractures primary skeletal survey	Occult fractures follow-up skeletal survey
0–6 (167)	96 (58)	57 (34)	110 (66)	73 (44)	16 (10)
7–12 (65)	35 (54)	15 (23)	39 (60)	9 (14)	
13–18 (20)	13 (65)	1 (5)	12 (60)	2 (10)	1 (5)
19–24 (20)	12 (60)	1 (5)	11 (55)	5 (25)	
25–36 (11)	9 (82)		6 (55)	2 (18)	
37–48 (12)	6 (50)		10 (83)	3 (25)	
Total (296)	172 (58)	74 (25)	189 (64)	94 (32)	17 (6)

The count and proportion (%) per age category of children is provided in the table (per subgroup)

The bottom line provides data on all children (*n* = 296) and % of total amount of children

Blank cells signify 0 subjects in this group

^a^Total number of subjects per category, the age of one child was unknown and is only included in the total amount

**Fig. 1:**
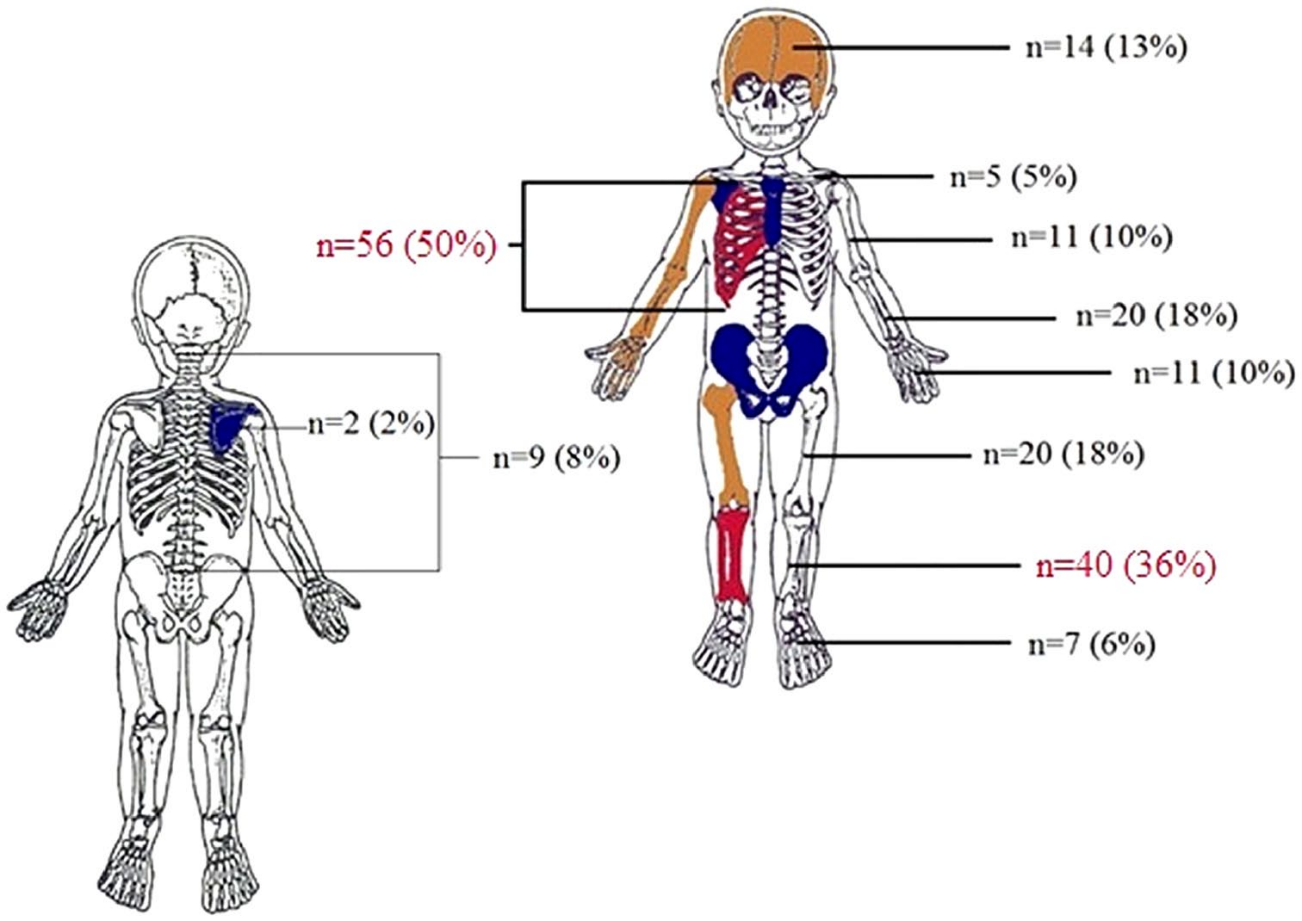
Prevalence and distribution of fractures (index fractures excluded). Total number of fracture location in children detected on the positive skeletal survey (primary and follow-up merged) and proportion (%) of the located fracture in all positive skeletal surveys (*n* = 111); e.g., in 56 children a rib fracture was detected, which is 50% of all children with a positive skeletal survey

The number of positive skeletal surveys per clinical injury stratified per age category are shown in Table 2 and per specific fracture as initial clinical injury in Table 3. The prevalence and distribution of occult fractures of all children are shown in Fig. 1. In our cohort 198 (67%) skeletal surveys were completed in accordance with the current guidelines.

**Table 2:** Positive skeletal survey per clinical injury

Age in months^a^	Fracture	AHT	Bruise	NAT sibling/twin	Total no. positive skeletal survey/total skeletal survey (%)
0–6	27/63 (43)	30/63 (48)	20/25 (80)	5/9 (56)	89/167 (53)
7–12	5/36 (14)	3/21 (14)	3/8 (38)	0/2 (0)	9/65 (14)
13–18	1/8 (13)		1/3 (33)	0/5 (0)	3/20 (15)
19–24	2/9 (22)		1/6 (17)	2/3 (67)	5/20 (25)
25–36	1/6 (17)		0/2 (0)	0/1 (0)	2/11 (18)
37–48	1/3 (33)		1/6 (17)	0/1 (0)	3/12 (25)
Total	37/126 (29)	33/90 (37)	26/50 (52)	7/21 (33)	111/296 (38)

Per age category and initial clinical injury for obtaining the skeletal survey is the count of positive skeletal survey versus the total skeletal surveys in this category provided and corresponding %. The column right provides data of all skeletal surveys per age category, positive skeletal survey count / total skeletal survey count and corresponding %

Blank cells signify 0 subjects in this group

*NAT* non-accidental trauma, *AHT* abusive head trauma

^a^Total number of subjects per category, the age of one child was unknown and is only included in the total amount

**Table 3 Positive skeletal survey per index fracture:** Number of subjects (%)

Age in months^a^	Fracture (total)	Upper extremity fracture	Lower extremity fracture	Skull fracture
0–6	27/63 (43)	7/9 (78)	11/24 (46)	2/14 (14)
7–12	5/36 (14)	1/7 (14)	2/14 (14)	2/13 (15)
13–18	1/8 (13)	0/1 (0)	1/6 (17)	0/1 (0)
19–24	2/9 (22)	2/5 (40)	0/3 (0)	0/1 (0)
25–36	1/6 (17)	0/2 (0)	1/4 (25)	
37–48	1/3 (33)	0/1 (0)	0/1 (0)	0/1 (0)
Total	37/126 (29)	10/25 (40)	16/53 (37)	4/30 (13)

Per age category and type of fracture (index fracture) as initial clinical injury for obtaining the skeletal survey is the count of positive skeletal survey versus the total skeletal surveys in this category provided and corresponding %. The left column provides data of all skeletal surveys per age category with a fracture as initial clinical injury (index fracture), positive skeletal survey count/total skeletal survey count and corresponding %

Blank cells signify 0 subjects in this group

^a^Total number of subjects per category, the age of one child was unknown and is only included in the total amount

In 94 (32%) of primary skeletal surveys occult fractures were detected and in 17 (6%) of all follow-up skeletal surveys. Long bone fractures were identified in 70 (24%) children, of which 11 (4%) humeral, 19 (6%) radial/ulnar, 20 (7%) femoral and 20 (7%) tibial/fibular fractures. Metaphyseal corner fractures were detected in 86 (29%) children. In 68 (23%) children acute and healing fractures were detected and in 35 (12%) children bilateral fractures. Data of type of fracture is stratified per age category in Table 4.

**Table 4:** Type of fracture on the skeletal survey

Age in months	MCF	Acute and healing	Bilateral fractures
0–6	80 (48)	60 (36)	28 (17)
7–12	3 (5)	6 (9)	4 (6)
13–18			
19–24	2 (10)	1 (5)	1 (5)
25–36	1 (9)		1 (9)
37–48	1 (8)	1 (8)	1 (8)
Total	86 (29%)	68 (23%)	35 (12%)

Amount of children stratified per age category and % of total amount of children of the subgroup

Blank cells signify 0 subjects in this group

*MCF* indicates metaphyseal corner fracture

**Fig. 2:**
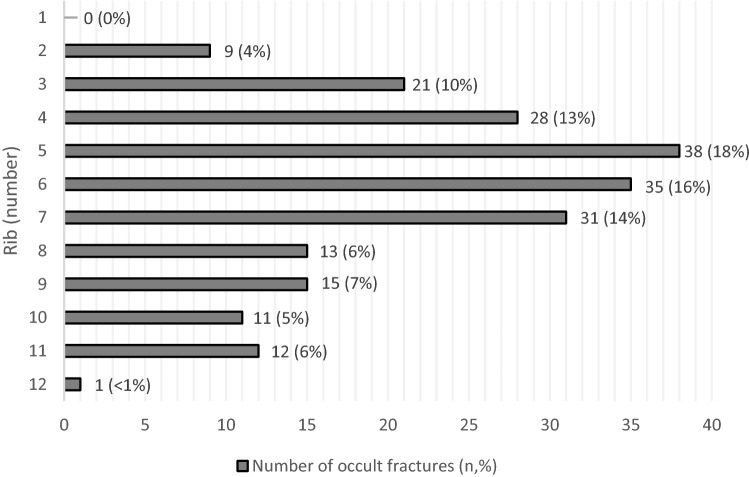
Prevalence and distribution of detected rib fractures on skeletal surveys (total rib fractures *n* = 216)

In total 216 rib fractures were detected on 49 skeletal surveys. Callus was visible in 37 primary surveys and follow-up skeletal surveys showed another seven children with rib fractures and callus formation. No fractures of the first rib were detected and only one fracture of the 12th rib was detected. Most frequently rib fractures were detected of the fourth up to the seventh rib (Fig. 2).

## Discussion

Our study demonstrates that occult fractures were detected in 32% of all children in our cohort who underwent a skeletal survey because of the evaluation of physical child abuse. Most prevalent detected occult fractures were located at the ribs and the tibia and fibula. We showed that the yield of the skeletal survey differs per clinical injury and age. The yield of the skeletal survey was the highest when the clinical injury suggestive for NAT for obtaining the skeletal survey was a bruise, suspected fracture or abusive head trauma (AHT), occult fractures were detected in 52%, 29% and 37% respectively.

### Prevalence and clinical injuries

The yield of the skeletal survey was higher in our cohort compared to other studies [6], however our cohort consisted mainly of high risk population. The yield of the skeletal survey in other studies ranged between 13% and 26% in infants and 7–19% in toddlers [6]. The prevalence of occult fractures in children imaged in our own hospital was 25% versus 35% in reviewed skeletal surveys (second opinion). Although our cohort consisted of relatively more second opinion cases, it shows the importance of the skeletal survey, as the yield in the high risk population is very high.

The high prevalence of occult fractures in children with AHT is in accordance with the literature [4, 8, 9]. Children with AHT are prone to suffer from non-accidental trauma, especially when they are young. Paine et al. showed similar results in their systematic review, with positive skeletal surveys in children with AHT ranging 23–34%. Isolated skull fractures had the lowest yield on skeletal surveys in their review (1–6%), which is in accordance with our results, we found occult fractures in 5% of children with an isolated skull fracture [6]. Further, occult fractures were frequently seen when the clinical injury was a suspected fracture in our cohort, especially when it was a long bone fracture. These results are comparable to the results of Barber et al. in their cohort of children with a suspected long bone fracture, the yield of the skeletal survey was 30% [4]. The number of children in our cohort with bruises was small and most of these children were younger than 6 months of age. Bruises in these young children without an apparent trauma should always raise suspicion [10]. In addition, we found a high percentage of positive skeletal surveys in children with bruises. Compared to Harper et al. they found occult fractures in 23% of young children (0–6 months) with bruises. These findings complement the current guidelines to obtain a skeletal survey in young children (< 1 year) with bruises [11].

Although the clinical reason ‘suspected non-accidental injury in a sibling/twin’ was not common in our cohort, occult fractures were very frequently detected, suggesting that siblings should be screened for occult injuries. Lindberg et al. reported a high prevalence of occult fractures among household contacts (siblings) of 11.9% in a large cohort [12]. Together with our findings this demonstrates the importance of obtaining a skeletal survey in (young) siblings and twins of abused children. Though we did not find a difference between siblings and twins, possibly because of the small group, twins seem to be at greater risk of having occult fractures than regular siblings and household contacts [12].

### Demographic characteristics

The majority of skeletal surveys were obtained in young children, aged younger than 6 months. The skeletal survey seemed to be an adequate diagnostic test to detect occult fractures in these young children. Although the skeletal survey was obtained infrequently in older children, we found a relatively high percentage of occult fractures among them. This suggests that even though older children have an improved ability to speak and express pain, clinically missed fractures were present. These findings suggest that the skeletal survey is valuable in older children as well. Lindberg et al. reported similar results and showed that the prevalence of occult fractures was similar between children aged 12–24 months (12%) versus children aged 24–36 months (10.3%) [13].

The skeletal survey is especially of great value in younger children since the quality of their bones differs from older children. Young children (< 12 months) have less strong and stiff bones than older children, but their bones are much more flexible compared to older children. The strength and stiffness increases with age, as demonstrated by Ambrose et al. [14]. This suggests that infants are more protected from getting fractured by external force than adolescents. Hence fractures in young children are harder to obtain and therefore these children should undergo a skeletal survey and an evaluation by a CAN—team. The protocol of the skeletal survey is based on the principle that the radiation risk should be as low as reasonably achievable. These findings support the guideline recommendation to perform a skeletal survey in young children and justify the radiation risk of this test. The estimated radiation dose of a skeletal survey in a child varies between 0.15 and 1.8 mSv, based on 21 images including oblique views of the ribs [5, 15–17]. In our opinion the yield of the skeletal survey outweighs the radiation risk of the skeletal survey, since this is up till now the most appropriate diagnostic tool to detect occult fractures.

### Distribution of occult fractures

Rib fractures were most prevalent detected occult fractures in our cohort. In accordance with the literature mid ribcage rib fractures were most common found [18, 19]. This suggests that the skeletal survey is pivotal in the detection of rib fractures, especially because these fractures can easily be missed on a regular chest X-ray [3, 4]. Incidental rib fractures are seldom detected as stated by Ruest et al. [20] They found an incidence of 0.05% incidental rib fractures in infants and toddlers who underwent a regular chest X-ray (for other reasons than NAT-evaluation). The obtained radiographs of the ribs are essential in the detection of rib fractures in children, which is endorsed in the guideline. Marine et al. showed an increased detection of rib fractures when a bilateral oblique rib radiograph was obtained. They found a sensitivity and specificity of 81–91% respectively, for the detection of posterior rib fractures [21]. Non-accidental injury should always be evaluated in young children with rib fractures, given the high specificity of rib fractures resulting from non-accidental trauma [22].

Long bone fractures were frequently detected, especially of the lower limb, which complements published literature. A greater proportion of lower limb fractures occur in young children, while upper limb fractures are more prevailing in older children [23].

The prevalence of pelvic, sternum and scapula fractures in our cohort was very low to none. This may suggest that these fractures are rarely caused by physical child abuse. This is shown by Lindberg et al. in a large cohort, they reported occult pelvic fractures in 0.3% of skeletal surveys and spine fractures in 0.8%, further Barber et al. reported 9.7% spinal fractures [24, 25]. This is expected since heavy forces are required in order to fracture the pelvis or sternum compared to limb fractures [26]. These findings suggest that the protocol of the skeletal survey should not be diminished to long bone and chest X-rays only, although pelvic, scapula, and sternum fractures are less common.

### Utilization of skeletal survey

We had to conclude that the skeletal survey was in 1/3 of all surveys not obtained in accordance with the current guideline [1, 2]. It is possible that occult fractures have been missed due to suboptimal images. Although our paediatric forensic radiologist recommended a follow-up skeletal survey, we did not receive the follow-up images of most referred cases. Further, in our own hospital not all children underwent a follow-up skeletal survey. We acknowledge that the follow-up skeletal survey requires coordination and effort to ensure this to be obtained 14 days after the initial skeletal survey. Nevertheless, the follow-up skeletal survey is pivotal in detecting occult fractures. Acute fractures of immature bones can be missed easily if soft tissue swelling is absent. The periosteal reaction develops usually after 5 days up to 2 weeks and soft callus after approximately 2 weeks [23]. Our results show that the guideline is not met in daily practice and follow-up skeletal survey utilization is not routinely performed, this is in accordance with the literature. The performance of follow-up of skeletal surveys ranged from 10–100% and identified new fractures in 8–28% [27]. Occult fractures were more likely to be detected in younger children and those with a fracture or cutaneous injury as clinical injury for the evaluation of child abuse [27].

### Imaging in older children

As stated above, the skeletal survey is pivotal for the detection of occult fractures, especially in young children. However, in some cases a bone scan is suggested for older children to identify old fractures. With respect to the indication for obtaining a bone scan in older children to detect occult fractures both the Royal College of Radiologists (RCR) and the American College of Radiologists (ACR) have made comments on this in their guidelines [1, 5]. The ACR Appropriateness Criteria^®^ does not include a statement regarding a bone scan in older children, but clarifies that a bone scan in young children (< 24 months old) is only indicated when the skeletal survey is negative and a high clinical suspicion remains [5]. The RCR states that “There is no evidence the bone scan will obviate the need for further imaging.” [1]. In addition, the bone scan cannot help with the dating of injuries, it can highlight areas of suspicion but further imaging is necessary to confirm whether there are any fractures [28]. Van Rijn and Sieswerda stated in their educational paper that another drawback of the bone scan is that there is a lack of experience in children, of which the consequence is a limited applicability because reading these studies will be insufficient [29]. On top of that the bone scan involves a high radiation dose compared to the skeletal survey (3 mSv versus 0.16 mSv effective dose) [5]. Therefore our advice is to obtain a skeletal survey if there is a (clinical) suspicion of NAT, even if the child is older than 24 months, because our data show that the yield of the skeletal survey in these children is sufficient and they will not be exposed to an unnecessary radiation risk.

### Implications of our findings

The high prevalence of occult fractures, which differs for specific clinical injuries, and distribution pattern of occult fractures in our cohort, complements published literature. Rib fractures were most prevalent, followed by fractures of the lower limb and although pelvic and sternum fractures were rare, the images of the skeletal survey protocol should not be diminished.

The high prevalence of occult injuries in children with bruises, fractures and AHT demonstrates the importance of the involvement of a paediatric surgeon in the evaluation of child abuse. This statement is endorsed by the American Pediatric Surgical Association [30]. The APSA advises a close cooperation of paediatric surgeons together with the CAN—team, especially because of the high possibility of polytrauma or severe injuries in these children.

### Strengths

These data demonstrate the distribution pattern of occult fractures in a large cohort of children with a high suspicion of being abused. We included all ages and showed that the skeletal survey is very valuable in children with bruises, fractures and AHT, however in older children and siblings as well. Another strength of this study was that all skeletal surveys are assessed by a specialized paediatric forensic radiologist.

### Limitations

A few limitations have to be addressed. Firstly, there is selection bias in our cohort. We only included suspected cases of non-accidental injury, in other words, the included skeletal surveys were part of the non-accidental injury work-up. Further, we included referred skeletal surveys for second opinion. This may have resulted in the inclusion of a relatively large group of abused children. Therefore these results are not applicable for the entire paediatric population. However, these data are valuable since they show the yield of the skeletal survey in a group of children with a high suspicion of child abuse. Lastly, there were less follow-up skeletal surveys in our cohort, therefore no strong conclusions can be drawn. A possible explanation is a lack of compliance to the protocols, however two third of our cohort was a request for second opinion. Though our specialised forensic radiologist recommended a follow-up skeletal survey, not all of those follow-up skeletal surveys were sent to our hospital. Unfortunately we do not have access to the reports of other hospitals and we were not able to check whether follow-up skeletal surveys were obtained.

## Conclusion

Occult fractures are frequently detected on the skeletal survey. When the clinical injury suggestive for NAT for obtaining a skeletal survey was a bruise, abusive head trauma or a long bone fracture, the prevalence of occult fractures was high. Most occult fractures were located at the ribs and tibia/fibula, while fractures located at the scapula, pelvis and sternum were infrequent. The low percentage of follow-up skeletal surveys in our cohort and compliance to the guidelines raise concern for underestimation and missed opportunities to detect occult fractures.

Although our data are obtained in a high risk population, the high prevalence of occult fractures endorses the use of a skeletal survey in the evaluation of child abuse.

## Abbreviations

NAT: Non-accidental trauma
AHT: Abusive head trauma
MCF: Metaphyseal corner fractures
CAN,: Child abuse and neglect team
